# Doing Genders: Partner’s Gender and Labor Market Behavior

**DOI:** 10.1177/00031224241252079

**Published:** 2024-05-22

**Authors:** Eva Jaspers, Deni Mazrekaj, Weverthon Machado

**Affiliations:** a Utrecht University; b University of Oxford

**Keywords:** labor market participation, household, sexual minorities, division of labor, gender

## Abstract

Partnered men and women show consistently gendered patterns of labor market behavior. We test whether not only a person’s own gender, but also their partner’s gender shapes hours worked. We use Dutch administrative population data on almost 5,000 persons who had both male and female partners, whose hours worked we observe monthly over 15 years. We argue that this provides a unique setting to assess the relevance of partner’s gender for labor market behavior. Using two-way fixed effects and fixed-effects individual slopes models, we find that both men and women tend to work more hours when partnered with a female partner compared to a male partner. These results align with our hypothesis that a partner’s gender influences labor market behavior. For women, we conclude that this finding may be (partly) explained by marital and motherhood status. Additionally, we discovered that women decrease their hours worked to a lesser extent when caring for a child if they have a female partner. Finally, we found that for men, the positive association between own and partner’s hours worked is weaker when one has a female partner, indicating a higher degree of specialization within these couples.

Despite an increase in their labor force participation over the past century, women still do a greater share of housework and spend less time in the labor market than do men (Charmes 2019). Even so, single men and single women show a much closer resemblance in the number of hours spent in paid work than do their partnered counterparts (Mazzocco, Ruiz, and Yamaguchi 2014; Ueno, Grace, and Šaras 2019). Maintaining a household requires financing but also domestic tasks, for example, grocery shopping, food preparation, and house cleaning. Unpartnered men and women perform all these tasks themselves, and although there is individual variation in how much time individuals spend on each one and whether they outsource such tasks, there is hardly any difference between unpartnered men and women in the number of hours spent in paid work. Once a person forms a joint household with a partner, however, sharp differences arise in time use: women bear the brunt of household tasks and childcare, while men spend more time in paid work.

In this article, we aim to reorient the literature, back to the original interactionist and relational understanding of gendered behavior. We take a unique approach to analyzing gendered labor market behavior and investigate whether individuals adjust their hours worked to their partner’s gender. To do this, we use Dutch administrative population data on almost 5,000 persons whose labor market outcomes we observed monthly over 15 years. These individuals were in committed relationships (cohabitation, registered partnerships, and marriages) with both male and female partners, allowing us to study changes in their hours worked. Using fixed-effects (FE) and fixed-effects individual slopes (FEIS) models, we ask whether persons who are in committed relationships with men and women adjust the number of paid hours they work to their partner’s sex.^1^ To the best of our knowledge, this is the first study to shed light on this question.

The gendered division of labor has traditionally been explained by specialization theories. For many reasons, be they gendered socialization, gender discrimination in the labor market, or gendered power dynamics, women typically find themselves with fewer marketable resources than their male partners. New home economics (Becker 1965) predicts that initial and minor differences in these resources translate into complete specialization over time, as specialization would be most beneficial to the household, although this is disputed by more recent work (Van der Vleuten, Evertsson, and Moberg 2023). The effects of economies of scale and household specialization work in opposite directions for men and women. A partnered woman will spend more time on housework than a single woman, and a partnered man will spend less time on housework than a single man. Doing a greater share of the housework comes at the expense of paid employment, resulting in less paid work for partnered women and more paid work for partnered men (Gupta 1999; Pepin, Sayer, and Casper 2018). However, this pattern has so far only been observed in different-sex couples (Evertsson and Boye 2018; Jaspers, Van der Lippe, and Evertsson 2022), as both male and female same-sex couples divide paid and unpaid labor more equally than do different-sex couples in both the United States (Brewster 2017) and Europe (Van der Vleuten, Jaspers, and Van der Lippe 2021).

Thus, although partners in same-sex couples may also have unequal marketable resources, these couples mostly do not show the same degree of specialization as different-sex couples. Several reasons for this difference have been proposed, such as non-heterosexual individuals having stronger equity beliefs (Shechory and Ziv 2007), but the “doing gender” theory has been most influential in explaining the division of labor for both different-sex and same-sex couples. According to this theory, gender is produced as a socially organized achievement and constituted through interaction (West and Zimmerman 1987). Women thus spend more time on housework and less time on paid labor as an act of “doing gender”: women, as well as the broader society, view domestic labor as an act that affirms femininity, and acting in accordance with gender norms comes with the rewards of fitting in.

For different-sex couples, a key component of affirming gender identity is by doing difference, that is, acts that are considered feminine can by definition not be masculine too (West and Fenstermaker 1995). For same-sex couples, gendered scripts of doing difference are absent; rather, these scripts would require each partner to behave similarly to both be affirmed in the same identity (Jaspers and Verbakel 2013). Same-sex couples thus divide housework and paid work more equally. Qualitative research indeed suggests that same-sex couples may negotiate the division of labor differently, without adhering as closely to gendered scripts, because one partner cannot enforce power over the other based on their gender alone. Research has described the negotiation process in same-sex couples as an opportunity for redoing gender by challenging normative gender roles (Kelly and Hauck 2015).^2^

We contribute to this literature by proposing that it is not only a person’s own gender that drives labor supply, as single men and single women display very similar patterns, but rather the interplay of one’s own gender, partner’s gender, and broader (societal) views on gender that shape expectations for how much time a person spends on paid work (Thomeer, Umberson, and Reczek 2020). Theoretically, we push the literature to once more regard gendered behavior as inherently and ultimately interactionist in nature, moving away from the simple dichotomy that can be found in the literature, wherein a person’s gender is assumed to drive behaviors by itself alone.

From an empirical standpoint, this is—to the best of our knowledge—the first article to analyze the relationship between a person’s hours worked and their partner’s gender. In doing so, we also contribute to the literature on people who form partnerships with both male and female partners.^3^ Although a large proportion of the LGBT+ population identifies as bisexual (Gates 2011), research on bisexual individuals is scarce and focuses primarily on mental health (see, e.g., Ross et al. 2018). Studies examining the labor market outcomes of bisexuals are rare. One important exception is Mize (2016), who used two survey datasets and showed that bisexual men and women are more disadvantaged in the labor market than gay men and lesbian women.

In the Netherlands, all employees have the right to request a reduction or increase in hours worked, and employers have only limited power to deny these requests. High-quality part-time jobs are readily available (Visser 2002), making this country an ideal site to test labor supply. Even so, disparities in hours worked between genders persist in the Netherlands, leading to a divergence in hourly wages: working fewer hours tends to yield lower wages compared to working more hours. As the first country in the world to legalize same-sex marriage, the Netherlands is a particularly useful setting for our study because of its long-standing recognition of same-sex partnerships and marriages (Lubbers, Jaspers, and Ultee 2009). The country also provides excellent access to longitudinal register data to study sexual minorities (Mazrekaj, De Witte, and Cabus 2020; Palmaccio, Mazrekaj, and De Witte 2023).

Our results indicate that both men and women work more hours when they are partnered with a female than with a male partner. Men even work more hours with a female partner than when they are unpartnered. This finding is in line with our hypothesis that a partner’s gender drives labor market behavior, above and beyond one’s own gender. Furthermore, we find that women who are caring for a child reduce their hours worked less when partnered with a woman, once again indicating that a partner’s gender affects individual labor market behavior. For men, we additionally observe that being partnered with a woman reduces the correlation between partners’ hours worked, suggesting more specialization in these couples.

## Theoretical Framework

### Expectations about Partners’ Doing of Gender

The division of paid and household labor allows women and men to show their femininity or masculinity by “doing gender” (Bianchi et al. 2012). According to West and Zimmerman’s (1987) pivotal work, it is essential that doing gender is understood as situated doing, carried out in the virtual or real presence of others who are presumed to be oriented toward its production. In their view, gender should be interpreted as something that emerges in social situations. Any participants in interactions “organize their various and manifold activities to reflect or express gender, and they are disposed to perceive the behavior of others in a similar light” (West and Zimmerman 1987:127). Goffman (1977) stressed that men and women strive to perform gender in accordance with others’ expectations. Women, then, would do more housework than men because they are held accountable to a higher degree for performing this feminine-typed task. The knowledge of others’ gendered expectations, even when they run counter to one’s own gender beliefs, may be enough to alter behavior. In her seminal work, Berk (1985) identified homes as “gender factories” in which gender is typically performed through continuous interactions.

Because of the interactionist nature of the performance of gender, a person reading a book on a couch in their home only does gender if we imagine their partner simultaneously vacuuming the floor under their feet. Departing from this perspective, we argue that even though a gender may be prescribed, and a gender role learned and internalized by individuals, it is in social situations that the performance of gender is expected and executed. We further argue that the extent to which individuals engage in doing or performing gendered labor depends on the gender of their partner in the interactions. An important feature of doing gender is that there is more than one gender, so certain actions and behaviors can be categorized as belonging to one, but not another, gender, in essence by “doing difference” (West and Fenstermaker 1995). Returning to the example above, a woman reading on the couch while her male partner vacuums would appear much more unusual than the other way around, as this particular situation goes against our gendered expectations of others’ behavior. If the vacuuming partner of the woman reading is another woman, however, the same scene is robbed of its gendered meaning, although the woman on the couch might be perceived as not fulfilling her proper feminine-typed task of cleaning (Carrington 1999; Thébaud, Kornrich, and Ruppanner 2021).

Especially for different-sex couples, (culture specific) gender discourses serve as frameworks for how partners perceive their relationship. Because these discourses frequently become entrenched within institutions, they encompass norms, values, and behaviors that dictate roles and responsibilities for each gender (Thomeer et al. 2020). Variation exists in the exact roles and responsibilities that are expected from men and women across educational categories (Budig, Lim, and Hodges 2021), but the division is always strongly gendered, with men prioritizing paid work. Generally, as education level rises, gender inequality decreases in areas such as labor force participation, hours worked, job segregation, and household tasks, as highly educated women have better employment opportunities. However, in the Netherlands, the gender wage gap is highest among highly educated individuals because men benefit more from education in this context (Evertsson et al. 2009).

From an interactionist doing gender perspective, individuals across successive different-sex unions would be doing gender by repeating the same division of labor they had established in their previous relationships (Ophir 2022). However, in the case of individuals who have both male and female partners, we posit that the interaction with partner’s gender influences how one performs gender and which behaviors are expected and deemed appropriate. In essence, these individuals are *doing genders* throughout their relationship trajectories. The gender of the partner in any interaction, but especially in committed relationships, raises expectations about the gendered doings of that partner and subsequent appropriate (gendered) responses to these expected behaviors. Both partners in two-person, coresidential, committed relationships do gender in relation to their partner’s gender in an effort to affirm both their own and their partner’s gender identity, and in more or less congruence with wider society-held beliefs on femininity and masculinity. Evidence outside the realm of the division of labor suggests individuals adjust their behavior in interaction with other people’s gender; for example, children in single-sex schools fare differently than children in mixed-gender schools in terms of gendered subject choice and girls’ lower sense of belonging (Brutsaert and Van Houtte 2002). Bisexual women have also been shown to adapt their looks—in a complex but very gendered way—to the gender of the person they are dating (Daly, King, and Yeadon-Lee 2018).

Individuals are prone to selective norm adherence when the exact nature of the norm is vague (Bicchieri 2016). For instance, regarding the norm that women should display caring behavior, it is not entirely clear what counts as caring and where one draws the line between caring and not caring. Previous research comparing different-sex and same-sex couples shows that compared to men, women provide more caregiving to their partner regardless of whether they are married to a woman or a man (Umberson et al. 2016), although in these studies the same individuals are never observed with both male and female partners. However, there is considerable variation within the groups of men and women in caregiving behavior.

An interactionist approach to doing gender, or the gender-as-relational approach (Thomeer et al. 2020), underscores the idea that caregiving is not singularly defined by an individual’s gender and that gendered dynamics within romantic relationships are collaboratively shaped by both one’s own gender and a partner’s gender. Consequently, it is not solely individual gendered experiences, norms, and beliefs that influence relationship dynamics, but also the gendered experiences of the partner. We extend this argument by stating that the behavior people believe another person would expect from them depends, in part, on who this other is. In our case, the other’s gender is the most important characteristic considered. When performing gender in a household with a partner, the doing gender expectation for a male partner is that they prioritize paid labor, whereas for a female partner, it is that they spend more time on housework. Naturally, these expectations work both ways, and gendered divisions of labor within the household are co-constructed in a process of constant (re)negotiation.

For the sake of clarity, in what follows, we will describe the process from the perspective of only one partner, as this aligns most with our empirical strategy. Thus, as the expected gendered behaviors of the partner inform the division of labor, we argue that having a female partner would mean spending more hours in paid work, and having a male partner would mean fewer hours to affirm the partner’s gender identity. Note that the exact process might work via different, yet related, mechanisms. For instance, one could prefer having a partner who performs gender appropriately as this might increase one’s own status (Pollitt, Robinson, and Umberson 2018). Alternatively, a partner could exert pressure to come to a division of labor that does not threaten their gender performance (Mannino and Deutsch 2007). Or third parties, such as employers, friends, or family members, may treat partners differently depending on the gender composition of the couple (Graham and Barnow 2013; Lloren and Parini 2017). We are unable to distinguish these mechanisms, but we argue they would all result in the hypothesized outcomes.

From a life-course perspective, family- and work-related outcomes are influenced by the timing of certain transitions (e.g., entering into a relationship), as well as by cumulative processes that build on previous biographic experiences and statuses (Bernardi, Huinink, and Settersten 2018; Elder, Johnson, and Crosnoe 2003). Furthermore, work and family trajectories are intertwined, which means events and states in these two life domains might be conceptualized as part of a broader multidimensional process (Aisenbrey and Fasang 2017). For example, within-couple inequality in the division of labor tends to increase over the course of (different-sex) relationships (Grunow, Schulz, and Blossfeld 2012). Given the gendered nature of the division of labor, this means women who spend a long time in committed different-sex relationships might have more incentives (or pressures) to work fewer hours. Moreover, labor market trajectories might affect whether, when, and with whom an individual enters into a partnership.

An individual’s labor market behavior and the characteristics—in our case, the sex—of their current partner can thus both be influenced by their past work and their family trajectories. Because we use a fixed-effects design, we rely on within-person variation in hours worked and, by definition, control for time-invariant attributes of individuals’ trajectories, such as the specific sequence of partnerships (e.g., having first a different-sex rather than a same-sex relationship). We incorporate relevant time-variant factors by controlling for the duration of the current relationship and the last spell without a partner, as well as the order of the partnership (e.g., first, second) in the individual’s life course.

Next, we turn to the issue of fertility. The arrival of children often results in a reshuffling of time spent on various tasks. Children require financial resources as well as more time spent on care. In different-sex couples, the first birth can be seen as the start of a strongly gendered division of labor from which women may never recover economically (Jaspers et al. 2022). The meaning of parenthood is clearly gendered in the Netherlands, with motherhood, in particular, performed according to a strong behavioral script (Machado and Jaspers 2023). However, the norm surrounding modern fatherhood is that young fathers, too, should spend time on childcare, especially among individuals who have not had solely heterosexual experiences (Roeters, Veerman, and Jaspers 2018). The Netherlands also facilitates part-time work and parental leaves for both parents, to some extent, irrespective of gender (Evertsson, Jaspers, and Moberg 2020). We thus assume that all parents in our sample dedicate some time to childcare and away from the labor market when there is a child in the house. When partnered with a woman, however, we anticipate that both men and women will not reduce their own hours as much, compared to when they are partnered with a man, to protect the female partner’s performance of gender through motherhood.

The dynamics of individual labor market behavior are, next to one’s own gender and other characteristics, contingent in part on other partner characteristics. On the one hand, from a financial perspective, having a partner who works very few hours is likely to increase the number of hours one spends in the labor market to secure sufficient family income. On the other hand, there has been a trend toward more assortative mating, with partnerships between individuals with similar status or earnings potential, which would suggest a positive association between partners’ time spent in paid labor (Vandecasteele and Esche 2016). Empirical research on different-sex couples in the Netherlands has found no evidence that the financial argument for specialization affected women’s hours worked (Verbakel 2010), potentially partly because of welfare state provisions, but other studies show a positive association between hours worked by men and fewer hours worked for women in different-sex couples (Brynin and Francesconi 2004).

We conclude that two forces may be at play. First, one of homogamy in terms of, for instance, education, level of ambition, and increased similarity due to shared lifestyle preferences as a relationship progresses. Second, having a female partner may drive a process of specialization, to safeguard the production of femininity for the female partner, increasing one’s own hours in paid work more strongly. Thus, we predict that a partner’s hours worked will be positively associated with a person’s own time in paid work. But, to protect a female partner’s feminine caregiving and homemaker identity, the positive association between one’s own and a partner’s paid hours worked should be less strong for men and women when they have a female partner compared to when they have a male partner.

### Hypotheses

*Hypothesis 1:* Both men and women spend more hours in paid labor when they have a female partner than when they have a male partner.
*Hypothesis 2a:* Children reduce the amount of time an individual spends on paid labor, but less so when their current partner is a woman.
*Hypothesis 2b:* A partner’s hours worked are positively associated with an individual’s own hours worked, but less so when the current partner is a woman.

## Data

### Sample Construction

We used longitudinal records collected by Statistics Netherlands that cover the entire population of individuals registered as residents in the Netherlands from 1995 to 2020. Each individual is assigned a unique identification number, allowing us to link information from various administrative registers. We started by examining the partnership registers, which gave us the personal identifier of each person’s partner and the dates on which the coresidential partnership began and ended. For people who are not married or in a registered partnership, identification as a couple by Statistics Netherlands is based on administrative and legal information that indicate a committed relationship. These indicators include notarial cohabitation agreements, being tax partners, being listed as partners in pension plans, and having a joint legal child, among others.^4^ Using the starting and ending dates for partnerships, we converted the dataset into a person-month dataset, as we knew from month to month with whom a person had a partnership. A person without a coresidential partner in a certain month was deemed “unpartnered.”^5^

We linked this dataset with demographic registers that include information on the date of birth, sex, and origin of each individual and their partner. This allowed us to identify individuals who had both male and female partners and whether they first had a male partner and then switched to a female partner or vice versa. We used household registers to determine the number of children living in the household, education registers to obtain partners’ education levels, and tax registers to obtain partners’ gross income. Finally, we linked our dataset with employment registers that include monthly information on the number of hours worked and gross wages of each person for the years 2006 to 2020.

Our administrative data offer three primary advantages over survey data. First, given that we monitor the entire population of the Netherlands for 15 years, we have a large enough sample of persons who had both male and female partners in the time frame studied. This is unprecedented in the literature, given that both a very large sample and longitudinal data over many years are necessary to identify individuals who had committed relationships with both male and female partners. Second, using administrative records minimizes measurement error because information on hours worked, wages, and sex was not reported by the individual but traced in employment and demographic registers. Survey data, in contrast, may be prone to misreporting and social desirability bias (Kreider and Lofquist 2015). Finally, we monitor highly detailed, month-by-month information rather than the usual annual data. This is beneficial because people can change their labor market behavior and partner several times within a year.

To study how a person’s labor market behavior varied with their partner’s sex, we restricted the sample in several ways. First, we considered the 2006 to 2020 period because not all labor market information we require is available before 2006.^6^ This gave us a large observation period of 180 months, or 15 years. We considered only individuals who resided in the Netherlands for this entire period (i.e., did not die or migrate within the observation window). Second, because we need variation in partner’s sex, we selected persons who had at least one male and one female partner within the observation period and lived with each of them for at least a year. We restricted the sample to individuals who were between 25 and 29 years of age in January 2006, and therefore age 40 to 44 by the end of the observation period (7,109 people). We chose the lower limit because we are interested in labor market behavior, and persons under age 25 are more likely to be students. By age 25, however, most people have typically entered the labor market. We chose the upper limit because we had information on union formation starting in 1995, whereas the labor market information only became available in 2006. A person who was 29 in 2006 would have been 18 in 1995, allowing us to trace the history of their unions from that age.

Next, we excluded data for years in which individuals were at least partially self-employed. Tax records provided data on whether individuals had earnings from self-employment in any given year, but we did not have information on the number of hours worked in self-employment. If an individual had both an employment contract—and thus appeared in the monthly employment register—and earnings from self-employment for the same year, we were unable to determine the total number of hours in paid work. Individuals who were self-employed the entire period were thus dropped from the sample, leaving us with 6,870 individuals. We further excluded partnerships that had large age gaps or involved family members of previous partners, as well as individuals who reunited with a previous partner after a cohabitation spell with a different partner.^7^ These partnerships might not have been romantic partnerships, and we apply the most stringent criteria. After these exclusions, we observed 5,210 people in two partnerships. Finally, we removed people with missing information or observations with implausibly large values on the outcome,^8^ which left us with the final sample of 4,972 persons (2,871 men and 2,101 women).

### Variables

Our dependent variable is monthly hours worked (including overtime), obtained from employment registers. Western countries, the Netherlands included, have prioritized reducing the conflict between work and family responsibilities and advancing gender equality by enhancing the compatibility of unpaid caregiving duties and paid employment, often through flexible working arrangements (Schmitt and Auspurg 2022). The option for shorter work hours, however, has intensified gender inequality by limiting career prospects, reducing work experience, and via employers linking flexible hours with reduced pay. Evidence from the United States indicates that widening gender disparities in working hours have impeded progress in narrowing the gender wage gap (Goldin 2014). Earlier studies show that welfare states promoting extensive female workforce participation through family-oriented policies can inadvertently exacerbate gender disparities (Mandel and Semyonov 2006). Indeed, increased flexibility in work hours contributes to perpetuating gender-based wage gaps in Germany (Schmitt and Auspurg 2022).

To summarize, given that work hours can be formally adjusted by individuals within the Dutch context, and given that work hours display strong gender associations and significantly contribute to later career gender disparities like hourly wages and income, our emphasis lies on labor supply as the primary outcome where individuals wield considerable influence to alter their personal outcomes in the short term. We bottom-coded this variable to zero, given that a small number of individuals had implausible values below zero. Months in which individuals did not have an employment contract and were not self-employed were assigned a value of zero in the analyses, for both working hours and the additional analyses we performed on wage.

Our main variable of interest is partner’s sex, constructed as an indicator with a value of 1 if the partner was female and 0 if the partner was male. Note that we have no information on the gender identity or sexuality of the individuals studied; we solely observe a person’s registered sex. In additional analyses, we used a trichotomous indicator for relationship type, namely female partner, male partner, and unpartnered. The latter category is defined as either single or in a non-coresidential relationship that we cannot detect in the data. For clarity, we use the term “unpartnered” throughout the manuscript.

In some specifications, we controlled for a continuous measure of the number of children in the household. This measure counts the number of children under 18 years of age and includes stepchildren. Patterns in the division of labor may be different in later unions. The few studies using longitudinal data point to remarkable stability across unions (Beblo and Solaz 2020; Ophir 2022), however, marriage (as opposed to cohabitation) usually increases within-couple inequality (Baxter, Hewitt, and Haynes 2008). Furthermore, (lengthy) spells of singlehood could lead to increases in hours worked that spill over into subsequent relationships. We controlled for a continuous measure of partner order that indicates the position of the current partner in the sequence of partners since the individual was 18 years old (1 is first partner, 2 is second partner, and so on). We also controlled for a binary indicator for the legal status of the union (1 is married or in a registered partnership, 0 is cohabiting), and continuous measures of the duration (in months) of the current relationship, and the duration (in months) of the last spell of living without a partner before the current relationship.^9^ Note that most of our sample is observed in only two coresidential relationships between 2006 and 2020. Specific relationship sequences (e.g., having a different-sex union first and then a same-sex union, versus the other way around) are a time-invariant attribute for most individuals. Figure S1 in the online supplement offers descriptive evidence that the differences in hours worked by partner’s sex have a similar pattern across relationship sequences.

Finally, in some specifications, we also use partner’s hours worked, measured in the same way as own hours worked, partner’s yearly gross income, and partner’s education. The latter is operationalized as an indicator with four categories: (1) *no high school diploma* (people with primary education and people who dropped out of secondary education without a high school degree), (2) *high school diploma* (people with a secondary education diploma or an associate degree that is not a bachelor’s degree, or people who dropped out of a bachelor’s program), (3) *bachelor’s degree* (either a college or a university bachelor’s degree, or people who dropped out of a master’s program), and (4) *master’s degree or PhD*.

## Methods

### Two-Way Fixed Effects

Given that we observe each person for up to 180 months, we used a two-way (individual and month/year) fixed-effects model. Essentially, we exploited variation in hours worked and partner’s sex within persons. The advantage of the fixed-effects model is that it controls for all time-invariant individual, unobserved characteristics related to hours worked and partner’s sex.^10^ The model can be formulated as follows:

(1) $$y_{it}=\beta_{1}S_{it}+\theta X_{it}+\gamma_{i}+\delta_{t}+\varepsilon_{it}$$

where *y_it_* represents paid hours worked of person *i* in month/year *t*. The variable of interest is *S_it_*, representing a binary indicator for a female versus a male partner. In some analyses, this variable is categorical instead of binary, as we added a third category that represents unpartnered observations. The parameter of interest is, representing the difference in hours worked when the partner is female versus male. Depending on the specification, Equation 1 also includes control variables defined above as part of ***X_it_***. In all specifications, we included individual fixed-effects and month/year fixed-effects . The individual fixed-effect subsumes all time-constant characteristics that affect a person’s hours worked in the same way over time. Time-varying variables that influence hours worked are captured by the error term *ε_it_*. We followed the advice of Cameron and Miller (2015) and clustered standard errors at the person level to account for the dependence of observations within persons.

It is useful to reflect on the conditions under which the parameter of interest represents the causal effect of partner’s sex on hours worked. First, there should be enough variation within each person. Given that our observation window covers up to 180 months and each person has had at least one male and at least one female partner by construction, this condition is likely to hold. Second, the partner’s sex should not be mismeasured. Our administrative data are based on automated municipal registers, so administrative errors are likely to be very small. From 2014, people in the Netherlands were allowed to legally change their sex on birth certificates and other official documents without undergoing sterilization or sex reassignment surgery. In our data, we considered an individual’s legal sex as it was in 2020, so any person—be it a focal individual or a partner—who legally changed their sex during or before our observation window was assigned for the whole period the sex they chose to have as their legal sex by then.^11^

A third condition is that there should be no reverse causality: a person’s hours worked should not influence their decision to be with a male versus a female partner. Although we are not aware of any research documenting this, we cannot be fully certain whether individuals who consider switching to a partner of a different gender than their current partner adjust their labor supply to become more attractive for the new potential partner. However, this does not refute our general thesis that partner’s gender and expectations about each person’s role in the division of labor in the household inform an individual’s labor supply. This factor would only alter the order of steps in the process; hence, we take this as a non-consequential condition.

As a final condition, the fixed-effects model requires strict exogeneity of the covariates: there should be no correlation between the error term and all past, current, and future time periods of the same person. In other words, there should be no unobserved factors that vary over time and are different between individuals. For people with both male and female partner trajectories, a change in the partner’s sex is inextricably linked with a change in the partner. Unobserved factors, such as a change in residence or a job change, may be linked to a change in the partner’s sex. To partially account for this, we conducted additional analyses in which we control for job change. Results in Table S4 in the online supplement are very similar to our main results in Table 2.^12^ In summary, although we cannot account for all unobserved factors that may coincide with a change in a partner’s sex, and therefore we caution the reader in interpreting our results as causal, we do provide the first evidence of how partner’s sex relates to a person’s labor market behavior.

### Fixed-Effects Individual Slopes

Assessing whether the relationship between children and partner’s hours worked and own hours worked is moderated by the sex of the partner requires the inclusion of interaction terms. Nonetheless, recent research shows that standard interaction estimates in fixed-effects models capture not only within-unit variation but also between-unit variation in the interacted variables (Giesselmann and Schmidt-Catran 2022). In our case, this would mean a standard interaction term between partner’s sex and number of children would not only capture how within-person variation in partner’s sex moderates the within-person association between having children and hours worked, but it would also capture how, for example, between-person variation in the mean of partner’s sex (i.e., proportion of months spent with a female partner) moderates the association between having children and hours worked.

We thus adopt one of the solutions proposed by Giesselmann and Schmidt-Catran (2022) and estimate fixed-effects individual slopes (FEIS) models including individual slopes for the interacted variables (see also Ludwig and Brüderl 2018; Rüttenauer and Ludwig 2020). FEIS models are also useful to address potential violations of parallel trends (Ludwig and Brüderl 2018).^13^ Essentially, the hours worked of people who switch from a male to a female partner and of people who are partnered with a male partner throughout may differ in level, but they should grow at the same rate. Currently, we lack studies that investigate whether this is in fact true. Thus, it is possible that persons are more likely to switch to a female partner than stay with a male partner if they are on a steeper trajectory in hours worked. FEIS models help address this concern.

## Results

### Sample Characteristics

Table 1 presents descriptive statistics for our sample, which consists of 4,972 individuals observed for a cumulative total of 461,253 months in which they lived with a partner.^14^ Except for the sex of the focal individual, all variables are time-varying. Women account for 42 percent of individuals. On average, women spend more months living with a partner than do men, as indicated by the variables duration of the current relationship and duration of the last spell of singlehood. Women are also more often in a formal relationship (married or registered partnership) and have more children living in the household.

**Table 1.** Descriptive Statistics

	Full Sample		Women		Men	
	Mean/%	Std. Dev.	Mean/%	Std. Dev.	Mean/%	Std. Dev.
	(1)	(2)	(3)	(4)	(5)	(6)
Age	34.16	4.57	34.28	4.59	34.06	4.55
Number of children in household	.86	1.04	.98	1.05	.77	1.02
Married/reg. partnership	37.0%		40.7%		34.3%	
Duration of current relationship (months)	51.47	41.09	54.42	42.73	49.27	39.68
Duration of last spell of singlehood (months)	46.31	41.26	44.65	38.95	47.55	42.87
Paid hours worked	121.05	63.61	109.37	59.30	129.77	65.30
Gross wage	2,671.93	2,500.33	2,357.49	2,002.78	2,906.55	2,791.88
Partner’s sex: female	58.8%		36.9%		75.1%	
Partner order	1.97	.75	1.92	.72	2.00	.76
Partner’s paid hours	104.13	69.92	119.49	68.14	92.66	69.03
Partner’s annual gross income	40,237.22	35,291.24	49,455.63	41,090.54	33,259.02	28,359.31
Partner’s education						
No high school diploma	19.0%		18.8%		19.2%	
High school diploma	23.9%		25.8%		22.5%	
Bachelor’s degree	30.2%		31.7%		29.1%	
Master’s degree / PhD	26.9%		23.8%		29.2%	
Individuals	4,972		2,101		2,871	
Observations	461,253		197,097		264,156	

Both men and women spent most of the observed period in different-sex relationships: men had a female partner in about 75 percent of the months observed, and women had a male partner in about 63 percent of months. This may partially reflect higher dissolution rates and shorter durations for same-sex unions. Research comparing union dissolution for same-sex and different-sex couples has produced mixed results, with some studies finding higher dissolution rates for same-sex couples and others finding no significant differences between couple types (Ketcham and Bennett 2019; Kolk and Andersson 2020; Manning, Brown, and Stykes 2016; Rosenfeld 2014). In the Netherlands, female same-sex marriages have higher divorce rates than do different-sex and male same-sex marriages (Kalmijn, Loeve, and Manting 2007; Statistics Netherlands 2021). There is also some evidence that individuals who identify as bisexual have more and lengthier different-sex relationships, and that they start such relationships at an earlier age than same-sex relationships (de Graaf and Picavet 2018).

The fact that most of our observations (person-months) refer to different-sex relationships is also relevant for interpreting the gap between average hours worked by the focal individuals and their partners. Men worked an average of 130 hours a month, and their (more often female) partners worked about 93 hours per month; on the other hand, women worked an average of 109 hours per month, and their (more often male) partners worked 119 hours per month. These averages include months in which individuals did not have a job, so they partially reflect employment rates. For reference, employed women in the Netherlands worked for a weekly average of 26 hours in 2020, and men worked for a weekly average of 34 hours (Statistics Netherlands 2023).

### A Person’s Hours Worked and Partner’s Sex

Table 2 presents models predicting monthly hours worked for the full sample and separately for men and women. All models include person and year/month fixed effects. The first three models (columns 1 to 3) show only the key predictor, the partner’s sex. The coefficients for this variable allow us to answer our main research question: does the labor market behavior of people who have relationships with both men and women depend on their partner’s sex? Our results suggest it does. In the full sample, having a female versus a male partner corresponds to a statistically significant difference of 16 hours of paid work per month. The difference is larger for men: men work 21 more hours in the months they have a female partner than when they are in a same-sex relationship. This amounts to about 16 percent of their average monthly hours (see Table 1). For women, the association is also positive and significant, albeit smaller in both absolute and relative terms: women work about seven more hours, about 6 percent of their average, when partnered with another woman. The models without controls thus show the pattern predicted by our first hypothesis: both men and women work more hours in paid work when they have a female partner.

**Table 2.** Fixed-Effects Models for Monthly Hours Worked

	Full Sample	Women	Men	Full Sample	Women	Men	Full Sample	Women	Men
	(1)	(2)	(3)	(4)	(5)	(6)	(7)	(8)	(9)
Partner sex (1 = female)	16.423***	6.692***	20.662***	16.987***	1.711	21.169***	17.771***	1.808	19.827***
	(1.033)	(1.423)	(1.498)	(1.031)	(1.442)	(1.533)	(1.046)	(1.547)	(1.632)
*N* children (stepchildren incl.)				−4.924***	−11.566***	−2.686***	−4.705***	−11.029***	−1.875*
				(.571)	(.827)	(.749)	(.598)	(.846)	(.809)
Married (1 = married/regist. partn.)				3.117**	−4.166**	3.995**	1.840	−3.893**	3.673**
				(1.006)	(1.383)	(1.415)	(1.020)	(1.412)	(1.421)
Partner high school diploma							1.002	4.168	−.465
(ref.: no high school diploma)							(2.311)	(3.326)	(3.135)
Partner bachelor’s degree							−2.155	−.811	−3.191
(ref.: no high school diploma)							(2.433)	(3.486)	(3.279)
Partner master’s/PhD							−4.547	−1.929	−5.792
(ref.: no high school diploma)							(2.688)	(3.842)	(3.627)
Partner paid hours worked							.060***	.043***	.058***
							(.006)	(.009)	(.008)
Duration of the current relationship							.068***	−.010	.052*
							(.017)	(.026)	(.025)
Duration of the last spell of singlehood							.003	.043	−.013
							(.019)	(.028)	(.025)
Partner order^a^							5.985***	1.242	2.947
							(1.560)	(2.529)	(2.074)
Partner gross income							.000	.000*	.000***
							(.000)	(.000)	(.000)
Observations	461,253	197,097	264,156	461,253	197,097	264,156	461,253	197,097	264,156
Individuals	4,972	2,101	2,871	4,972	2,101	2,871	4,972	2,101	2,871

*Note:* Standard errors clustered at the individual level are in parentheses. All models are estimated using individual and year/month fixed effects. Sample: people with both male and female partners between 2006 and 2020, age 25 to 29 at the beginning of the panel. The coefficient of interest is the coefficient on “partner sex,” which can be interpreted as follows: if a woman is with a female partner (column 2), the monthly number of hours worked increases by 6.692 hours compared to when a woman is with a male partner.

a Position of the current partner in the sequence of partners since the individual was 18 years old (1 = first partner, 2 = second partner, and so on).

* *p* < 0.05; **p < 0.01; ***p < 0.001 (two-tailed *t*-tests).

In a second set of models (columns 4 to 6 in Table 2), we consider two family characteristics that are relevant for labor force participation and the division of labor in couples, namely the legal status of the relationship (marriage/registered partnership versus cohabitation) and number of children in the household (including stepchildren). Inclusion of these controls barely changes the association between partner’s sex and hours worked for men, but it decreases the association for women, making it statistically insignificant, albeit still positive. This pattern persists when we further control for partner’s education and hours worked, partner’s gross income, partner order, duration of the relationship, and duration of the last spell of singlehood (columns 7 to 9). Overall, these results suggest the sex of the partner matters for the hours worked of women primarily as a correlate of having children and being in a formal relationship, two key predictors of female paid labor supply (see, e.g., Bianchi et al. 2012; Verbakel 2010).^15^ It is consistent with the argument that women may have less gendered scripts prior to being wives and mothers, with the full force of gender norms only experienced once they enter these statuses. Working fewer hours in paid labor only then becomes part of the normative female identity.

Table 3 presents the results of models predicting hours worked for all months for which we have valid information (using the same sample of individuals), regardless of whether the focal individuals were living with a partner. These models allow us to compare hours worked by individuals across three different partnership states: living with a male partner (the reference category), living with a female partner, and not living with a partner. We do not expect the contrast between being with a male or a female partner to be different here, but it is still useful to consider how not living with a partner compares to living with a male or a female partner. On average, persons in our sample spent 27.2 percent of months with a male partner, 38.8 percent with a female partner, and 33.9 percent unpartnered. The results are very similar to those described above. In models without controls, men and women work the least when they are partnered with a man—the coefficients for being unpartnered and for having a female partner are both positive and statistically significant. The absolute difference between being unpartnered and having a male partner is similar for men (about 9 hours per month) and women (about 7.8 hours). When controlling for number of children in the household, differences become smaller for women but remain essentially unaltered for men.

**Table 3.** Fixed-Effects Models for Monthly Hours Worked, Singlehood Spells Included

	Full Sample	Women	Men	Full Sample	Women	Men
	(1)	(2)	(3)	(4)	(5)	(6)
Female partner (ref.: male partner)	17.016***	6.579***	21.457***	17.901***	2.629*	22.442***
	(.948)	(1.304)	(1.363)	(.950)	(1.276)	(1.383)
Unpartnered (ref.: male partner)	8.441***	7.755***	8.977***	7.407***	3.158**	8.994***
	(.831)	(1.104)	(1.210)	(.826)	(1.092)	(1.210)
*N* children (stepchildren incl.)				−4.021***	−11.531***	−1.326*
				(.515)	(.756)	(.662)
Observations	698,100	294,753	403,347	698,100	294,753	403,347
Individuals	4,972	2,101	2,871	4,972	2,101	2,871

*Note:* Standard errors clustered at the individual level are in parentheses. All models are estimated using individual and year/month fixed effects. Sample: people with both male and female partners between 2006 and 2020, age 25 to 29 at the beginning of the panel. The coefficient of interest is the coefficient on “female partner,” which can be interpreted as follows: if a woman is with a female partner (column 2), the monthly number of hours worked increases by 6.579 hours compared to when a woman is with a male partner. Similar interpretation holds for the coefficient on “unpartnered.”

* *p* < 0.05; **p < 0.01; ***p < 0.001 (two-tailed *t*-tests).

Returning to the analysis of months when individuals were partnered, recall that in Hypotheses 2a and 2b we posited that children in the household would reduce the focal individual’s hours worked, whereas the partner’s hours worked would increase those of the focal individual, and that both of these associations would be weaker when the partner is a woman. The results presented in Table 2 indeed show that children are associated with working fewer hours, and that the individual’s and their partner’s hours worked are positively correlated. Women’s hours worked are more affected by children than are men’s hours, which is consistent with the extensive literature documenting gender inequality in the consequences of parenthood.^16^

Next, we assess whether the association between children and partner’s hours worked and own hours worked is moderated by the sex of the partner. As previously mentioned, we use FEIS models for this purpose and the results are presented in Table 4. Models in columns 1 to 3 include the interaction between partner’s sex and number of children while allowing for individual-specific slopes for these two variables, hence the absence of common slopes for them in the table. A positive coefficient for the interaction term indicates that the (within-person) association between children and hours worked is less negative when someone has a female rather than a male partner. In other words, having a female partner seems to weaken the detrimental effect of parenthood, which is consistent with Hypothesis 2a. This finding echoes previous research comparing same-sex and different-sex couples but, to the best of our knowledge, this is the first time this pattern has been documented based on within-person variation.

**Table 4.** Fixed-Effects Individual Slopes (FEIS) Models for Monthly Hours Worked, with Interactions

	Full Sample	Women	Men	Full Sample	Women	Men
	(1)	(2)	(3)	(4)	(5)	(6)
Partner sex x Children	14.598***	18.443***	−2.108			
	(3.308)	(3.808)	(5.403)			
Partner sex x Partner paid hours worked				−.043**	.009	−.042*
				(.015)	(.022)	(.019)
*N* children (stepchildren incl.)				−6.623***	−13.299***	−2.164**
				(.594)	(.854)	(.761)
Partner paid hours worked	.031***	.026***	.029***			
	(.005)	(.007)	(.007)			
Married (1 = married/regist. partn.)	.422	−3.327*	4.019**	.421	−3.143*	3.708**
	(1.057)	(1.447)	(1.513)	(1.017)	(1.353)	(1.424)
Partner high school	7.070	6.560	7.324	−2.256	10.548	−8.919
(ref.: no high school diploma)	(5.119)	(9.170)	(6.196)	(5.619)	(10.181)	(6.294)
Partner bachelor’s degree	−6.148	−3.978	−7.818	−9.666	.945	−13.649*
(ref.: no high school diploma)	(6.628)	(13.168)	(7.292)	(6.701)	(13.557)	(6.858)
Partner master’s/PhD	−2.751	.911	−4.234	−11.031	1.076	−16.343*
(ref.: no high school diploma)	(7.211)	(13.740)	(8.254)	(6.592)	(12.686)	(7.236)
Duration of the current relationship	.006	−.036	.044	−.020	−.053	.026
	(.037)	(.059)	(.047)	(.036)	(.054)	(.044)
Duration of the last spell of singlehood	.003	.036	−.014	−.002	.065	−.034
	(.034)	(.064)	(.040)	(.034)	(.055)	(.040)
Partner order^a^	3.079	−1.252	5.533	−.455	−2.324	.865
	(2.371)	(4.191)	(2.841)	(2.357)	(3.915)	(2.802)
Partner gross income	.000	.000	.000*	.000*	.000	.000
	(.000)	(.000)	(.000)	(.000)	(.000)	(.000)
Observations	461,253	197,097	264,156	461,253	197,097	264,156
Individuals	4,972	2,101	2,871	4,972	2,101	2,871

*Note:* Standard errors clustered at the individual level are in parentheses. All models are estimated using individual and year/month fixed effects. Sample: people with both male and female partners between 2006 and 2020, age 25 to 29 at the beginning of the panel. The coefficients of interest are interactions between partner’s sex and number of children (columns 1, 2, and 3), and between partner’s sex and partner’s hours worked (columns 4, 5, and 6). Our specification included individual slopes for the variables involved in each interaction, thus there are no overall coefficients capturing the main effects. A positive interaction between partner’s sex and number of children means the association between children and hours worked is more positive (or, equivalently, less negative) when individuals have female partners rather than male partners. A similar interpretation holds for the interaction between partner’s sex and partner’s hours worked.

a Position of the current partner in the sequence of partners since the individual was 18 years old (1 = first partner, 2 = second partner, and so on).

* *p* < 0.05; **p < 0.01; ***p < 0.001 (two-tailed *t*-tests).

Nonetheless, as the models in columns 2 and 3 show, this moderation relationship is only positive and statistically significant for women. Not only are men’s hours worked less affected by the presence of children (as discussed above), but the association is similarly small whether these men are in different- or same-sex relationships, indicating it is indeed both own and partner’s sex that matter in labor market decisions. The performance of paid work is more central to masculinity, and more core to broader societal gendered expectations for men as breadwinners, making it no surprise men are affected more strongly in their hours worked by their partner’s gender and less by the presence of children. For women the opposite may be true: motherhood is such a central part of femininity that women’s hours worked may be more strongly affected by the presence of children than by their partner’s gender. Moreover, the likelihood of having children is much higher with a different-sex partner.

Finally, columns 4 to 6 of Table 4 show analogous models, now focusing on the interaction between partner’s sex and hours worked, thus testing Hypothesis 2b. Once more, we find that the moderation hypothesis is only partially confirmed, in this case only for men. All the previous models that include a main effect for partner’s hours worked show it is positively associated with one’s own hours worked, so the negative coefficient for the interaction term indicates that, for men at least, this association is weaker when the partner is female, pointing at more within-household specialization.

In the online supplement, we report additional models for hours worked with the sample split by whether an individual was first observed in a different-sex or a same-sex relationship (Tables S2 and S3), as well as models for monthly gross wages (Table S6). Across these models, the coefficients for having a female partner are similar to those in Table 2: they are consistently positive and statistically significant for the full sample and for men, and are generally smaller—and, in some cases, not significant—for women, especially after controlling for motherhood and marital status. Figure S2 shows the results of fixed-effects models predicting the change in hours worked from the first to the second relationship for all persons who were 25 to 29 years in 2006 and are observed with at least two partners (only different-sex, only same-sex, or a combination of both) during our observation period. All these results align with our main conclusions and provide a stronger case for the observed differences in labor supply being driven by partner’s sex rather than respondents’ unobserved characteristics.

## Discussion

Our study consistently showed that it is not only an individual’s own gender but also their partner’s gender that contributes to labor supply. We obtained our results using a unique design that allowed us to observe within-person differences in behavior depending on the partner’s sex and involving data on 4,972 Dutch individuals with both male and female partners over a 15-year period. It appears that the sharp difference between straight and gay and lesbian households in labor market behavior that is becoming a stylized fact in the literature (Machado and Jaspers 2023) may not be explained by selection into specific relationships alone. We have shown that individuals who have committed relationships with both women and men display systematically different behavior with their female than with their male partners. Labor market behavior should thus be understood as arising not only from the gender of an individual but also from the gender of their partner.

We theorized that this pattern is due to an orientation toward producing not only one’s own gender identity by performing gender-typed tasks, but also to the partner’s gender identity by protecting their production of gender-typed tasks. This double orientation works both ways and leads to the continuous co-production of genders within a relationship. To the best of our knowledge, we are the first to show that the same individuals perform their gender depending on their partner’s gender, thereby “doing genders.” In doing so, we hope to have reoriented theorizing about gendered behavior to the original interactionist and situational understanding, especially within the realm of the household, one of the main “factories” where gender is produced, in line with the classical understanding of doing gender (West and Zimmerman 1987) and more recent explorations of the gender-as-relational framework (Thomeer et al. 2020).

Our findings tentatively indicate that a partner’s gender may play a significant role in women’s hours worked mainly due to its association with having children and being in a formal union, both of which are crucial factors influencing female participation in paid labor. For women, gendered scripts are stronger when they are wives and mothers. We observed a stronger association between hours worked and partner’s sex for men than for women, which may be because paid work is more central to doing gender for men.

We ruled out a number of alternative explanations that might lead to the same empirical observations. First, an important reason for working more hours might be having a lower-paid partner. In the Netherlands and elsewhere, women continue to earn less than men (Christofides, Polycarpou, and Vrachimis 2013), so the earnings of female partners—earnings which are themselves tied to broader societal or workplace gender norms—might increase an individual’s own labor supply. However, our models for labor supply patterns controlled for partner’s gross annual income. This indicates that the mechanism driving the increase in hours worked when having a female partner is not related to a partner’s lower earnings. As argued before, flexibility in hours worked also perpetuates gender-based wage gaps and vice versa (Schmitt and Auspurg 2022). Given that hours worked can be formally adjusted by individuals within the Dutch context, and hours worked display strong gender associations and significantly contribute to later career gender disparities like hourly wages and income, we maintain that labor supply is the primary outcome to study to understand partner influence on personal labor market decisions.

Second, we know that individuals may receive differential treatment from employers, dependent on their sexuality (Drydakis 2022). A change in partner might coincide with a change in employer (e.g., when one moves to a different location to be with the new partner), so part of the difference in hours worked might be because of the sexuality an employer perceives. Employers might offer more hours to men who have a female partner than men who have a male partner. Even though these effects might be limited, and any such differential treatments would be more likely to appear in hourly wages, we ran additional analyses where we controlled for change of employer (reported in Table S4 in the online supplement). Our results remained unaltered. Hence, we have no indication that labor market discrimination is driving our results. We also controlled for the potential effect of “coming out” on these pathways. Sexual orientation can evolve, and within these trajectories, some individuals (including lesbian and gay persons) may transition to same-sex relationships after disclosing their sexual orientation. However, Figure S1 in the online supplement shows that our results are robust for relationship pathways from different-sex to same-sex or vice versa.

The present study is not without limitations. First, we analyzed individuals with both male and female partners, meaning we cannot infer with certainty that what we observed would hold for individuals who form committed relationships with only one sex. Individuals with both male and female partners may differ inherently, in various aspects, from people consistently in different-sex or same-sex relationships. For instance, individuals experiencing or destined for relationships with both male and female partners might adopt a more adaptable lifestyle, less bound by fixed routines, which could manifest in their ability to adjust hours worked more readily throughout their lives. The work hour patterns observed in this study’s sample might therefore be linked to an overall flexibility in lifestyle, or other particularities of the work and family experiences of sexual minorities. Indeed, research has established more complex family trajectories, worse mental health, and worse labor market outcomes for bisexual individuals (Mize 2016; Ophir, Boertien, and Vidal 2023; Pompili et al. 2014).

Given the nature of our data, we cannot determine the sexual orientation nor the gender identity of the individuals in our sample, which might include, for example, bisexual, queer, and pansexual people, as well as gay and lesbian people who come out later in life. The extent to which our results apply to all these different groups—and how well they generalize to people with more conventional relationship trajectories—is hard to ascertain empirically and predict theoretically. Nonetheless, we maintain that our findings help explain why the gendered division of labor shows such a stubborn pattern once joint households are formed, in line with the argument that studying sexual minorities also helps explain gendered behavior in heterosexual couples (Evertsson, Kirsch, and Geerts 2021).

Second, although much of the literature on the gendered division of labor focuses on the division of housework, we were unable to do so in the present study. We had no choice but to focus on masculine-typed behavior that is registered in official Dutch statistics, which might help explain the somewhat larger coefficients we observed for men. We argue that it is highly likely that the same gendered process is related to stereotypically feminine-typed tasks in the household as well. We urge others to think of ways to test for the performance of household tasks and care among individuals who have committed relationships with both men and women.

Third, even though we may theoretically speculate how a partner’s gender relates to an individual’s hours in paid labor, we cannot test the actual mechanisms. For instance, we cannot deduce whether it is the partner who exerts pressure to conform to gendered scripts to protect their own gender identity, or whether the behavior to protect a (romantic) partner’s gender performance is internalized and driven by individuals themselves. It is also possible, or even likely, that a part of the mechanism through which doing genders operates is via the couple’s wider network—including employers—who may treat people differently depending on the gender composition and broader societal gender scripts that are enacted within a couple. Furthermore, gender is associated with power, with men typically having a higher social status than women. Gendered status beliefs contribute to a framework of limited options and social responses for women in the context of masculine-typed tasks. These existing distinctions in status and authority have consequences in social interactions. Individuals reinforce and perpetuate existing gender roles and hierarchies through their daily interactions, thereby contributing to the maintenance of the gender system. In settings involving both genders, these status beliefs may influence the probability of assuming breadwinner roles for men and women differently (Ridgeway 2001; Ridgeway and Smith-Lovin 1999).

Finally, as previously mentioned, we urge caution in interpreting our results as causal. We cannot completely dismiss the possibility that individuals contemplating a change from a partner of one gender to another may modify their participation in the labor market to appeal more to their (potential) new partner. Nevertheless, it is important to acknowledge that this does not disprove our overall thesis, which suggests a (future) partner’s gender and their assumptions about paid labor division can influence one’s own labor supply. It would simply reorganize the sequence of actions within this process.

We would encourage future replications in other countries, as the Dutch context is rather specific, with an ample availability of good-quality part-time jobs and flexibility to change one’s number of hours worked. In contexts where it is harder to adjust hours worked according to family circumstances, or where there is (legally and symbolically) a stronger divide between part-time and full-time work, partner’s gender might have a weaker influence on one’s (changes in) paid work, with “doing gender” being more restricted to the division of housework. The poorer mental health that people with both male and female partners may experience, due, for instance, to stigma or greater likelihood of rejection from both the straight and gay communities (Pompili et al. 2014), may also affect the number of hours spent in paid labor. This is beyond the scope of the present study but would be a highly relevant way forward for future research.

We present only means for hours worked across all months spent with (male and female) partners, but future studies might investigate how exactly partner’s hours influence and are influenced by an individual’s hours over time. It is beyond the current article’s scope to address these linkages over the duration of the relationship, but it would greatly enhance our understanding of how gender (in)equality in paid work is produced within families from a life-course perspective. We note there is a scarcity of theorizing about and observations of the longitudinal dynamics of household divisions of labor, apart from the well-established shocks of parenthood, unemployment, and retirement. Finally, much can be learned from studying this group of individuals who form relationships with both male and female partners, for instance, when it comes to fertility and relationship duration and divorce. We would therefore encourage scholars to take up these and other topics for individuals with both male and female partners.

## Supplemental Material

sj-pdf-1-asr-10.1177_00031224241252079 – Supplemental material for Doing Genders: Partner’s Gender and Labor Market Behavior Supplemental material, sj-pdf-1-asr-10.1177_00031224241252079 for Doing Genders: Partner’s Gender and Labor Market Behavior by Eva Jaspers, Deni Mazrekaj and Weverthon Machado in American Sociological Review

## Notes

1. Although we acknowledge more than two genders and a variety of gender expressions, and we are aware people may have relationships with more than one gender for various reasons, we have no information on the gender identity nor sexuality of the individuals studied. We can only identify a person’s registered sex in our administrative data and ascertain whether individuals have had relationships with both men and women. Whether they themselves would identify as bisexual, gay, queer, pansexual, straight, or otherwise is unknown to us.

2. However, other work suggests that men and women in same-sex relationships still need to maintain their gender identities by doing, or not doing, housework (Oerton 1997, 1998). For instance, Blumstein and Schwartz (1983) found that both straight and gay men viewed performing household labor as demasculinizing, and both men in gay relationships expected their partner to perform paid labor.

3. As we do not know the sexual orientation of the individuals in our study, we refrain from identifying them as bisexual. Instead, we describe the pattern of their relationships we can observe, and thus refer to them as individuals with both male and female partners.

4. It is possible that people live together as a couple but are not identified as such by Statistics Netherlands (and thus are not included in our sample) because they lack any administrative or legal tie that indicate a committed partnership. Except for relatively short relationships, these cases should be rare, as Statistics Netherlands also applies the identification criteria retrospectively: if two people start living together and after some time, even years later, information denoting a committed partnership between them (e.g., having a child) emerges, their partnership is considered to have begun when they registered as residents at the same address.

5. Note that these individuals are not necessarily single. For instance, a person may be in a relationship but not live at the same address as their partner. Given that we were only interested in partnerships with a shared household, it was unnecessary to distinguish between single persons and persons in a non-coresidential relationship.

6. Although the COVID-19 pandemic started in 2020, our results are virtually unchanged if we exclude 2020 from the sample. Hence, we opted to include 2020 for a larger sample size.

7. Namely, we excluded relationships in which (a) the (absolute) age gap between the partners is larger than 25 years; or (b) the partner is a family member (parent, grandparent, sibling, niece/nephew, uncle/aunt) of any previous partner of the individual. The latter criterion accounts for cases of temporary living arrangements with in-laws that might be erroneously classified as a partnership. We also excluded (c) rare cases in which the partner is an extended family member (e.g., first cousin) of the individual; or (d) individuals who have two separate spells of cohabitation with partner A, with a spell of cohabitation with partner B in-between. In a large share of these cases, partner A is a different-sex partner, partner B is a same-sex partner, and there is no or only a short time gap between the relationships with A and B. This might suggest the cohabitation period with partner B is erroneously classified by Statistics Netherlands as a partnership. Note that all of these cases might still encompass real partnerships, as register data capture even relatively rare family patterns (e.g., partnering with a cousin of your ex), but we believe these restrictions significantly reduce ambiguities in the identification of couples. Nonetheless, our results are robust using a sample without these restrictions.

8. In the Netherlands, a person can generally work a maximum of 60 hours per week, amounting to 270 hours (60 hours multiplied by 4.5 weeks) a month.

9. As a robustness check, we also estimated models using the cumulative count of months living without a partner since the individual’s 18th birthday, instead of the duration of last spell. The results, which are virtually the same as our main analyses, are shown in Table S5 in the online supplement.

10. Note also that including age in the model is not necessary because of the two-way fixed effects.

11. We used the legal sex in 2020 instead of before 2014 because the number of persons who changed their legal sex in our sample was negligible, and the results when excluding these individuals were virtually the same.

12. Note that hours worked could change with the same employer if people feel less comfortable at work when, for instance, having a same-sex partner.

13. This added benefit of FEIS is why we prefer it over the other solution proposed by Giesselmann and Schmidt-Catran (2022), namely the double-demeaned estimator. Nonetheless, we obtain similar results with both strategies.

14. Table S1 in the online supplement presents the descriptive statistics by sex and relationship trajectory.

15. Note, however, that some of the control variables may be endogenous to partner sex. For instance, it is likely easier to have children in a different-sex rather than a same-sex relationship (Mazrekaj et al. 2020). Thus, the number of children may be influenced by partner’s sex and therefore endogenous. This may in turn lead to post-treatment bias (Rosenbaum 1984), also known as “collider bias” (Elwert and Winship 2014) or “bad control bias” (Angrist and Pischke 2009).

16. Although we do acknowledge that interpreting control variables may be prone to the Table 2 fallacy (Westreich and Greenland 2013).
