# Supplementary material for: Doing Genders: Partner’s Gender and Labor Market Behavior
Source: Am Sociol Rev. 2024 May 22;89(3):518–41. doi: 10.1177/00031224241252079 (PMC11144571; doi:10.1177/00031224241252079)
Supplement: sj-pdf-1-asr-10.1177_00031224241252079 – Supplemental material for Doing Genders: Partner’s Gender and Labor Market Behavior [file sj-pdf-1-asr-10.1177_00031224241252079.pdf]

AMERICAN SOCIOLOGICAL REVIEW

OFFICIAL JOURNAL OF THE AMERICAN SOCIOLOGICAL ASSOCIATION

ONLINE SUPPLEMENT

to article in

AMERICAN SOCIOLOGICAL REVIEW, 2024, VOL. 89

Doing Genders: Partner's Gender and Labor Market Behavior

Eva Jaspers
Utrecht University

Deni Mazrekaj
Utrecht University and University of Oxford

Weverthon Machado
Utrecht University

TABLE S1. DESCRIPTIVE STATISTICS, BY SEX AND RELATIONSHIP TRAJECTORY

	Women, started w/ different-sex relationship		Women, started w/ same-sex relationship		Men, started w/ different-sex relationship		Men, started w/ same-sex relationship	
	Mean/%	Std. Dev.	Mean/%	Std. Dev.	Mean/%	Std. Dev.	Mean/%	Std. Dev.
	(1)	(2)	(3)	(4)	(5)	(6)	(7)	(8)
Age	34.45	4.62	34.10	4.55	34.02	4.60	34.08	4.53
Number of children in the household	1.05	1.08	.91	1.01	0.65	.97	.82	1.03
Married/reg. partnership	43.4%		37.8%		30.3%		35.8%	
Duration of current relationship (months)	55.96	44.99	52.72	40.02	41.99	36.80	52.16	40.41
Duration of last spell of singlehood (months)	40.41	35.88	49.35	41.58	42.66	41.89	49.48	43.10
Paid hours worked	106.14	60.11	112.94	58.18	114.76	75.14	135.71	59.94
Gross wage	2,131.49	1,835.61	2,607.58	2.145.27	2,086.14	2,043.17	3.231.53	2.975.96
Partner's sex: female	50.4%		22.1%		63.5%		79.7%	
Partner order	1.93	.81	1.91	.62	2.08	.99	1.97	.64
Partner's paid hours worked	115.90	68.55	123.46	67.47	85.11	72.59	95.66	67.34
Partner's annual gross income	42,925.39	30,445.30	56,682.24	49,312.86	27,339.23	22,860.77	35,753.54	29,929.47
Partner's education								
No high school diploma	24.9%		12.0%		35.1%		12.8%	
High school diploma	31.9%		19.0%		32.5%		18.6%	
Bachelor's degree	28.3%		35.3%		20.6%		32.4%	
Master's degree / PhD	14.9%		33.7%		11.8%		36.1%	
Individuals	1,153		948		988		1,883	
Observations	103,537		93,560		74,948		189,208	

TABLE S2. FIXED-EFFECTS MODELS FOR MONTHLY HOURS WORKED, STARTED WITH A DIFFERENT-SEX RELATIONSHIP

	Full sample	Women	Men	Full sample	Women	Men	Full sample	Women	Men
	(1)	(2)	(3)	(4)	(5)	(6)	(7)	(8)	(9)
Partner sex (1 = Female)	9.395***	7.575***	12.934***	10.596***	3.092	10.431***	11.270***	2.402	8.084*
	(1.505)	(2.164)	(2.460)	(1.538)	(2.277)	(2.907)	(1.623)	(2.760)	(3.171)
N children (stepchildren incl.)				–3.894***	–8.686***	–0.539	–3.708***	–8.255***	.599
				(.909)	(1.160)	(1.486)	(.948)	(1.168)	(1.533)
Married (1 = married/regist. partn.)				1.372	–4.470**	8.626**	.682	–3.917*	8.427**
				(1.457)	(1.731)	(2.816)	(1.566)	(1.791)	(2.949)
Partner high school (ref.: dropout)							1.061	1.145	–.215
							(2.810)	(3.880)	(4.009)
Partner bachelor's degree (ref.: dropout)							.162	–3.844	5.093
							(3.083)	(4.164)	(4.686)
Partner master's/PhD (ref.: dropout)							–4.613	–6.495	–1.810
							(3.870)	(4.983)	(6.225)
Partner paid hours worked							.057***	.040***	.073***
							(.009)	(.012)	(.014)
Duration of the current relationship							.026	–.044	.063
							(.024)	(.031)	(.043)
Duration of the last spell of singlehood							.074*	.080	.041
							(.029)	(.042)	(.042)
Partner order[a]							2.509	–3.909	6.235*
							(2.144)	(2.853)	(3.167)
Partner gross income							.000	.000*	.000
							(.000)	(.000)	(.000)
Observations	178,485	103,537	74,948	178,485	103,537	74,948	178,485	103,537	74,948
Individuals	2,141	1,153	988	2,141	1,153	988	2,141	1,153	988

Note: Standard errors clustered at the individual level are in parentheses. All models are estimated using individual and year/month fixed effects. Sample: people with both male and female partners between 2006 and 2020, age 25 to 29 at the beginning of the panel. The coefficient of interest is the coefficient on "partner sex," which can be interpreted as follows: if a woman is with a female partner (column 2), the weekly number of hours worked increases by 7.575 hours compared to when a woman is with a male partner.

[a]Position of the current partner in the sequence of partners since the individual was 18 years old (1 = first partner, 2 = second partner, and so on).

**p* < 0.05; ***p* < 0.01; ****p* < 0.001 (two-tailed *t*-tests).

TABLE S3. FIXED-EFFECTS MODELS FOR MONTHLY HOURS WORKED, STARTED WITH A SAME-SEX RELATIONSHIP

	Full sample	Women	Men	Full sample	Women	Men	Full sample	Women	Men
	(1)	(2)	(3)	(4)	(5)	(6)	(7)	(8)	(9)
Partner sex (1 = female)	20.331***	–1.921	15.513***	20.507***	2.150	15.260***	20.228***	3.556	13.122***
	(1.388)	(2.509)	(2.203)	(1.374)	(2.486)	(2.194)	(1.372)	(4.050)	(3.149)
N children (stepchildren incl.)				–6.900***	–12.919***	–3.756***	–6.280***	–12.967***	–2.812**
				(.792)	(1.313)	(.944)	(.813)	(1.294)	(.972)
Married (1 = married/regist. partn.)				–.534	–4.437	.959	–.785	–5.151*	1.074
				(1.349)	(2.330)	(1.622)	(1.338)	(2.262)	(1.605)
Partner high school (ref.: dropout)							1.700	12.808*	–2.709
							(3.924)	(6.040)	(4.936)
Partner bachelor's degree (ref.: dropout)							–3.340	7.776	–8.267
							(3.886)	(6.000)	(4.797)
Partner master's/PhD (ref.: dropout)							–3.255	9.015	–8.928
							(4.021)	(6.115)	(5.040)
Partner paid hours worked							.049***	.044***	.049***
							(.008)	(.012)	(.009)
Duration of the current relationship							.058*	.103*	.043
							(.026)	(.043)	(.032)
Duration of the last spell of singlehood							–.017	.031	–.031
							(.025)	(.040)	(.031)
Partner order[a]							5.709*	9.456*	3.917
							(2.224)	(4.784)	(3.025)
Partner gross income							.000	.000	.000**
							(.000)	(.000)	(.000)
Observations	282,768	93,560	189,208	282,768	93,560	189,208	282,768	93,560	189,208
Individuals	2,831	948	1,883	2,831	948	1,883	2,831	948	1,883

Note: Standard errors clustered at the individual level are in parentheses. All models are estimated using individual and year/month fixed effects. Sample: people with both male and female partners between 2006 and 2020, age 25 to 29 at the beginning of the panel. The coefficient of interest is the coefficient on "partner sex," which can be interpreted as follows: if a man is with a female partner (column 3), the weekly number of hours worked increases by 15.513 hours compared to when a man is with a male partner.

[a]Position of the current partner in the sequence of partners since the individual was 18 years old (1 = first partner, 2 = second partner, and so on).

*$p < 0.05$; **$p < 0.01$; ***$p < 0.001$ (two-tailed *t*-tests).

TABLE S4. FIXED-EFFECTS MODELS FOR MONTHLY HOURS WORKED, CONTROLLING FOR JOB CHANGE

	Full sample	Women	Men	Full sample	Women	Men	Full sample	Women	Men
	(1)	(2)	(3)	(4)	(5)	(6)	(7)	(8)	(9)
Partner sex (1 = female)	16.423***	6.692***	20.662***	16.982***	1.687	21.175***	17.766***	1.788	19.829***
	(1.033)	(1.423)	(1.498)	(1.032)	(1.442)	(1.533)	(1.046)	(1.548)	(1.632)
N children (stepchildren incl.)				–4.923***	–11.565***	–2.687***	–4.703***	–11.027***	–1.875*
				(.571)	(.827)	(.749)	(.598)	(.846)	(0.809)
Married (1 = married/regist. partn.)				3.127**	–4.156**	4.001**	1.849	–3.884**	3.679**
				(1.007)	(1.383)	(1.416)	(1.020)	(1.413)	(1.421)
Changed job (1 = yes)				2.000**	2.501**	2.200**	2.144***	2.510**	2.292**
				(.608)	(.898)	(.798)	(.602)	(.893)	(0.793)
Partner high school (ref.: dropout)							1.000	4.170	–0.468
							(2.311)	(3.326)	(3.135)
Partner bachelor's degree (ref.: dropout)							–2.154	–.813	–3.188
							(2.433)	(3.485)	(3.279)
Partner master's/PhD (ref.: dropout)							–4.546	–1.926	–5.790
							(2.689)	(3.842)	(3.627)
Partner paid hours worked							.060***	.043***	.058***
							(.006)	(.009)	(.008)
Duration of the current relationship							.068***	–.010	.052*
							(.017)	(.026)	(.025)
Duration of the last spell of singlehood							.003	.043	–.013
							(.019)	(.028)	(.025)
Partner order[a]							6.003***	1.257	2.962
							(1.561)	(2.531)	(2.073)
Partner gross income							.000	.000*	.000***
							(.000)	(.000)	(.000)
Observations	461,253	197,097	264,156	461,253	197,097	264,156	461,253	197,097	264,156
Individuals	4,972	2,101	2,871	4,972	2,101	2,871	4,972	2,101	2,871

Note: Standard errors clustered at the individual level are in parentheses. All models are estimated using individual and year/month fixed effects. Sample: people with both male and female partners between 2006 and 2020, age 25 to 29 at the beginning of the panel. The coefficient of interest is the coefficient on "partner sex," which can be interpreted as follows: if a woman is with a female partner (column 2), the weekly number of hours worked increases by 6.692 hours compared to when a woman is with a male partner.

[a]Position of the current partner in the sequence of partners since the individual was 18 years old (1 = first partner, 2 = second partner, and so on).

*$p < 0.05$; **$p < 0.01$; ***$p < 0.001$ (two-tailed *t*-tests).

TABLE S5. FIXED-EFFECTS MODELS FOR MONTHLY HOURS WORKED, CONTROLLING FOR CUMULATIVE TIME WITHOUT A PARTNER

	Full sample	Women	Men	Full sample	Women	Men	Full sample	Women	Men
	(1)	(2)	(3)	(4)	(5)	(6)	(7)	(8)	(9)
Partner sex (1 = female)	16.423***	6.692***	20.662***	16.987***	1.711	21.169***	17.960***	2.099	19.553***
	(1.033)	(1.423)	(1.498)	(1.031)	(1.442)	(1.533)	(1.050)	(1.587)	(1.651)
N children (stepchildren incl.)				–4.924***	–11.566***	–2.686***	–4.589***	–11.022***	–1.693*
				(.571)	(.827)	(.749)	(.599)	(.851)	(.818)
Married (1 = married/regist. partn.)				3.117**	–4.166**	3.995**	1.671	–3.992**	3.701**
				(1.006)	(1.383)	(1.415)	(1.022)	(1.415)	(1.421)
Partner high school (ref.: dropout)							1.045	4.151	–.330
							(2.318)	(3.347)	(3.129)
Partner bachelor's degree (ref.: dropout)							–2.105	–.587	–3.046
							(2.436)	(3.505)	(3.267)
Partner Master's/PhD (ref.: dropout)							–4.456	–1.757	–5.644
							(2.689)	(3.859)	(3.613)
Partner paid hours worked							.059***	.042***	.058***
							(.006)	(.009)	(.008)
Duration of the current relationship							.095***	–.003	.088**
							(.020)	(.029)	(.029)
Cumulative number of months unpartnered (since 18th birthday)							.126***	.053	.123*
							(.038)	(.053)	(.054)
Partner order[a]							6.071***	.187	3.788
							(1.474)	(2.394)	(1.999)
Partner gross income							.000	.000*	.000***
							(.000)	(.000)	(.000)
Observations	461,253	197,097	264,156	461,253	197,097	264,156	461,253	197,097	264,156
Individuals	4,972	2,101	2,871	4,972	2,101	2,871	4,972	2,101	2,871

Note: Standard errors clustered at the individual level are in parentheses. All models are estimated using individual and year/month fixed effects. Sample: people with both male and female partners between 2006 and 2020, age 25 to 29 at the beginning of the panel. The coefficient of interest is the coefficient on "partner sex," and can be interpreted as follows: if a woman is with a female partner (column 2), the weekly number of hours worked increases by 6.692 hours compared to when a woman is with a male partner.

[a]Position of the current partner in the sequence of partners since the individual was 18 years old (1 = first partner, 2 = second partner, and so on).

*$p < 0.05$; **$p < 0.01$; ***$p < 0.001$ (two-tailed t-tests).

TABLE S6. FIXED-EFFECTS MODELS FOR MONTHLY GROSS WAGES

	Full sample	Women	Men	Full sample	Women	Men	Full sample	Women	Men
	(1)	(2)	(3)	(4)	(5)	(6)	(7)	(8)	(9)
Partner sex (1 = female)	367.669***	–32.330	543.892***	347.678***	–74.915	368.637***	431.219***	–26.195	440.466***
	(29.024)	(40.018)	(40.541)	(28.800)	(41.964)	(42.392)	(29.283)	(48.975)	(44.974)
N children (stepchildren incl.)				61.367***	–122.495***	120.459***	24.630	–130.150***	88.770**
				(18.199)	(23.618)	(25.123)	(18.700)	(23.241)	(27.042)
Married (1 = married/regist. partn.)				264.933***	–1.324	350.109***	194.849***	–11.187	290.201***
				(32.525)	(39.859)	(50.105)	(34.183)	(41.396)	(51.286)
Partner high school (ref.: dropout)							–20.525	24.594	–5.034
							(48.801)	(68.470)	(67.133)
Partner bachelor's degree (ref.: dropout)							–76.028	14.338	–159.148*
							(57.156)	(81.297)	(75.027)
Partner master's/PhD (ref.: dropout)							–248.208***	–145.192	–324.403***
							(70.700)	(102.763)	(91.675)
Partner paid hours worked							.568**	.602**	–.119
							(.193)	(.232)	(.239)
Duration of the current relationship							1.709**	–.377	.524
							(.588)	(.675)	(.988)
Duration of the last spell of singlehood							–1.083*	–.659	–.962
							(.545)	(.838)	(.697)
Partner order[a]							–83.529	–65.710	–277.992***
							(45.416)	(61.913)	(65.325)
Partner gross income							.005***	.002	.010***
							(.001)	(.001)	(.001)
Observations	461,253	197,097	264,156	461,253	197,097	264,156	461,253	197,097	264,156
Individuals	4,972	2,101	2,871	4,972	2,101	2,871	4,972	2,101	2,871

Note: Standard errors clustered at the individual level are in parentheses. All models are estimated using individual and year/month fixed effects. Sample: people with both male and female partners between 2006 and 2020, age 25 to 29 at the beginning of the panel. The coefficient of interest is the coefficient on "partner sex," and can be interpreted as follows: if a man is with a female partner (column 3), the monthly gross wage increases by 543.892 euros compared to when a man is with a male partner.

[a]Position of the current partner in the sequence of partners since the individual was 18 years old (1 = first partner, 2 = second partner, and so on).

*$p < 0.05$; **$p < 0.01$; ***$p < 0.001$ (two-tailed *t*-tests).

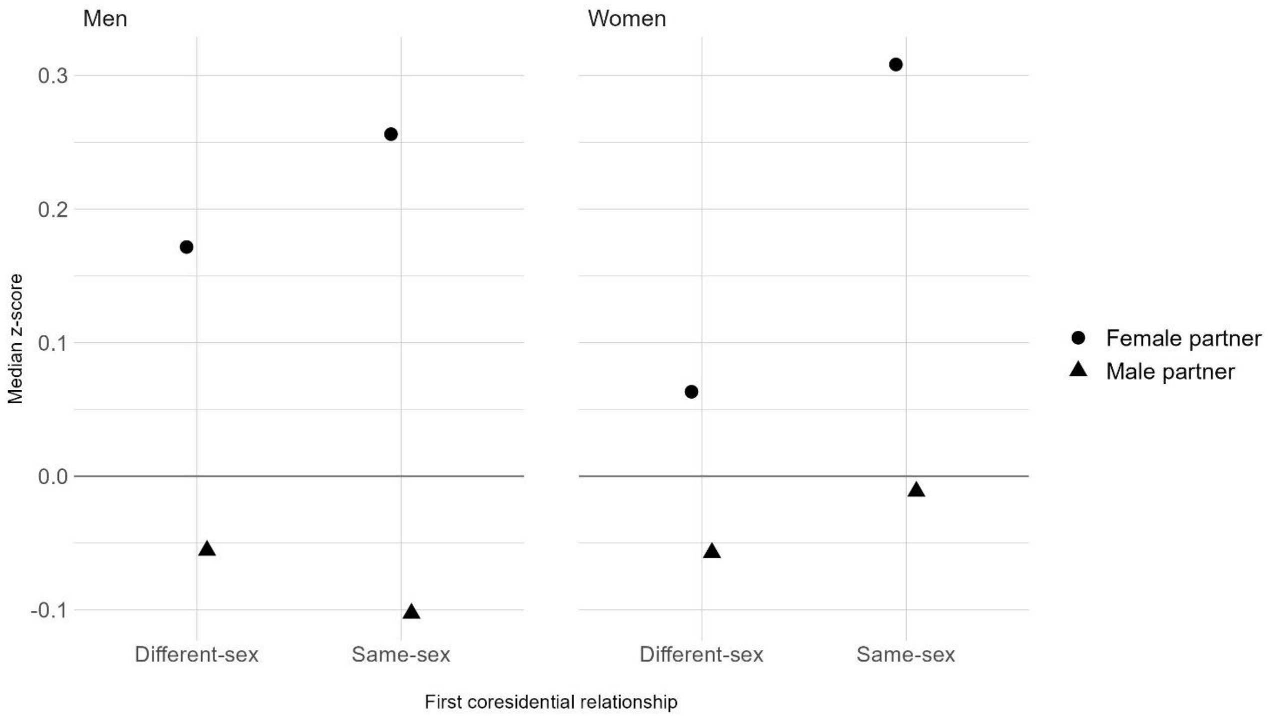

FIGURE S1. MEDIAN Z-SCORE OF MONTHLY HOURS WORKED, BY SEX, TYPE OF FIRST RELATIONSHIP AND PARTNER'S SEX

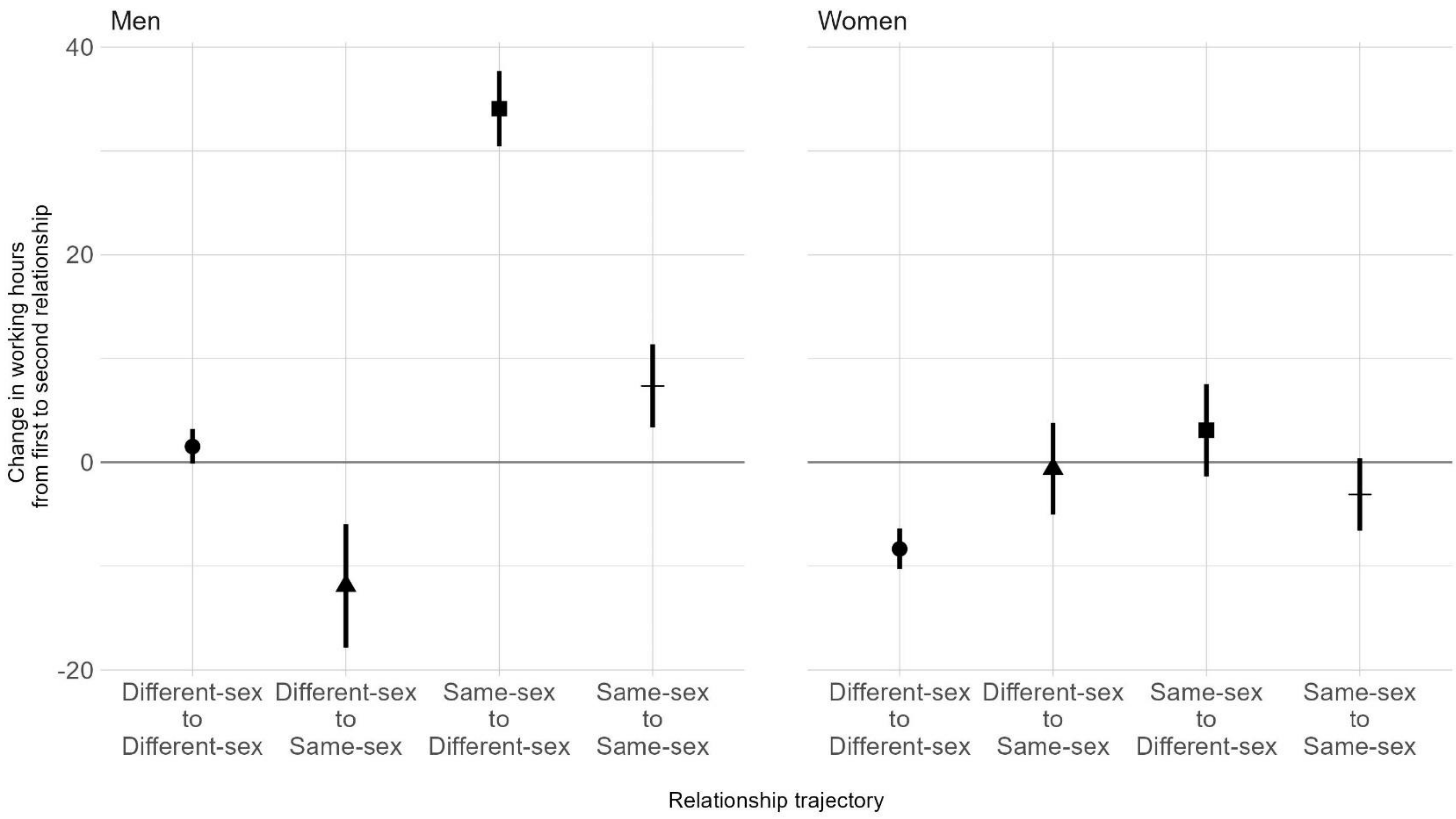

FIGURE S2. CHANGE IN HOURS WORKED FROM THE FIRST TO THE SECOND RELATIONSHIP, BY SEX AND RELATIONSHIP TRAJECTORY

Note: The underlying sample consists of persons who were 25 to 29 years in 2006 and are observed in at least two relationships during our observation period (2006 to 2020), regardless of whether these relationships are with a partner of the same or different sex. In other words, these individuals are not only the ones in our main sample—who have both male and female partners—but also individuals with *only* male or *only* female partners. In this analysis, we disregard any partnerships after the first two for each individual. We used fixed-effects models to estimate the change in hours worked from the first to the second relationship, while controlling for different variables: number of children, married/registered partnership, duration of current relationship, duration of previous singlehood spell, partner's education, partner's hours worked, and partner's gross income. Note that the trajectory of the first two relationships (e.g., different-sex to different-sex, different-sex to same-sex, and so on) is a time-constant attribute of the individuals, but trajectory-specific estimates for change in hours worked are obtained with interaction terms.

Obtaining Access to the Data from Statistics Netherlands

The data used in this study include detailed information on the entire population of the Netherlands and are therefore highly confidential. To access these data, a researcher must submit a proposal that outlines the objectives of the project as well as the datasets the researcher deems necessary. The full data catalog can be found here: https://www.cbs.nl/en-gb/our-services/customised-services-microdata/microdata-conducting-your-own-research/microdata-catalogue. Each project must include a researcher who is affiliated with a research institution in the Netherlands and most of the individual data files are in Dutch only. The project costs vary and depend mainly on the duration of the project, the number of researchers, and the data files requested. More information about the costs can be found here: https://www.cbs.nl/en-gb/our-services/customised-services-microdata/microdata-conducting-your-own-research/services-and-costs.

Once the project is approved, the researchers must pass a test administered by Statistics Netherlands that includes 20 questions on what actions are allowed in terms of privacy issues. Upon passing the test, the authorized researchers can access the data both within and outside the Netherlands through a high-security remote-access environment. Note that the results can only be used outside this environment after they have been approved and inspected by Statistics Netherlands for potential privacy breaches. For additional questions about data access, please contact Statistics Netherlands at the following email address: microdata@cbs.nl.

We consider the replication and validation of published research to be very important. Hence, we have made the codes available at the following link: https://osf.io/qpa8d. We would be happy to aid in obtaining access to the data and replicating the paper.
